# LocustDB: a relational database for the transcriptome and biology of the migratory locust (*Locusta migratoria*)

**DOI:** 10.1186/1471-2164-7-11

**Published:** 2006-01-21

**Authors:** Zongyuan Ma, Jun Yu, Le Kang

**Affiliations:** 1 National Laboratory of Integrated Management of Pest Insects and Rodents, Institute of Zoology, Chinese Academy of Sciences, Beijing 100080, China; 2 Beijing Genomics Institute, Chinese Academy of Sciences, Beijing Airport Industrial Zone-B6, Beijing 101300, China

## Abstract

**Background:**

The migratory locust (*Locusta migratoria*) is an orthopteran pest and a representative member of hemimetabolous insects for biological studies. Its transcriptomic data provide invaluable information for molecular entomology and pave a way for the comparative research of other medically, agronomically, and ecologically relevant insects. We developed the first transcriptomic database of the locust (LocustDB), building necessary infrastructures to integrate, organize, and retrieve data that are either currently available or to be acquired in the future.

**Description:**

LocustDB currently hosts 45,474 high-quality EST sequences from the locust, which were assembled into 12,161 unigenes. It, through user-friendly web interfaces, allows investigators to freely access sequence data, including homologous/orthologous sequences, functional annotations, and pathway analysis, based on conserved orthologous groups (COG), gene ontology (GO), protein domain (InterPro), and functional pathways (KEGG). It also provides information from comparative analysis based on data from the migratory locust and five other invertebrate species, including the silkworm, the honeybee, the fruitfly, the mosquito and the nematode. The website address of LocustDB is .

**Conclusion:**

LocustDB starts with the first transcriptome information for an orthopteran and hemimetabolous insect and will be extended to provide a framework for incorporating in-coming genomic data of relevant insect groups and a workbench for cross-species comparative studies.

## Background

Studying genetics and ecology of the evolutionarily diversified insects at molecular level is the most exciting area of entomology research today [1]. Acquisitions of sequencing data and their comparative analyses build a foundation for understanding biological pathways, molecular processes, and gene expression patterns, which are all relevant to physiological and genetic mechanisms of development, behavior, immunity, and phenotypic plasticity of the insects [2,3]. Efforts to acquire and integrate transcriptomic and genomic data are initial yet essential steps, and we have done so now by adding invaluable transcriptomic information from a new insect order, *Orthoptera*, to the existing data involving three other insect orders, *Lepidoptera, Diptera*, and *Hymenoptera*.

Our data came from a study concerning the migratory locust (*Locusta migratoria*), a representative member of hemimetabolous insects, which has a unique behavioral phenotype: changing phases from a solitary state to a gregarious one when environmental and genetic factors interact due to crowdedness [4]. Given the importance of studying locust as one of the major agricultural pests, we have developed a comprehensive and high-quality database, LocustDB (the Locust Database), for integrating, organizing, and retrieving sequences and related information. LocustDB provides a permanent platform for comparative studies of biology, genetics, and evolution of the locust. It currently hosts a large collection of expression-sequence-tags (EST), unigenes, and their annotations, and integrated comparative analysis results from five other invertebrate species whose genomic information has become available from large-scale genomic studies, including the silkworm, the honeybee, the fruit fly, the mosquito, and the nematode. LocustDB is the first genomic database for a hemimetabolous insect of orthopterans.

## Construction and content

### Data acquisition

EST sequences were generated from two types of cDNA libraries, the organ-specific and the mixed. The first is composed of six non-normalized, uni-directionally cloned cDNAs made from mRNAs of heads, hind legs, and midguts of fifth-instar locusts in two phenotypic phases: solitary and gregarious. The mixed library was constructed with mRNAs from the whole-body of the gregarious locust. Clones from these libraries were sequenced from the 5'-ends.

### EST assembly and gene annotation

We developed a data mining pipeline that analyzes EST data from multiple resources. The software package, Phred-Phrap-Consed, was used for base-calling, quality assessment, and sequence assembly [5,6]. Poly (A) tails, low quality data, and vector sequences were screened by CROSS_MATCH, and removed from the dataset. Sequences less than 100 bp in length were also discarded. A total of 45,474 high-quality ESTs with an average length of 471 bp were assembled with stringent Phrap parameters, yielding 12,161 contigs. Redundant mitochondrial RNAs, rRNAs, and *E. coli *contaminations were eliminated from the final assemblies.

We carried out a comprehensive annotation procedure for the locust unigenes. The clustered unigenes were annotated, based on a series of blast-based homology analysis [7]: (1) BLASTN versus NCBI's non-redundant nucleotide database, (2) BLASTX (E-value less than 1E-5) versus NCBI's non-redundant protein database, and (3) BLASTX versus the non-redundant protein database from SWISS-PROT. Unigenes were annotated with Gene Ontology (GO) terms by comparing the sequences against the database. Sequences with significant matches and best hits were classified according to the database's classification schemes [8]. We also compared our contigs and singlets using RPS (Reverse PSI) BLAST [9,10] to sequences of the COG (conserved orthologous genes) database and assigned the corresponding unigenes into COG functional classifications [11]. Functional domains from non-redundant sequences were assigned based on information from InterPro database [12]. Pathway analysis was performed against KEGG database with BLAST (Release 33) [13]. In addition, we compared the unigenes with genome data from the silkworm, the honeybee, the fruit fly, the mosquito, and the nematode to further define orthologous genes in Ensembl [14] and SilkDB [15].

### Implementation

LocustDB was organized with a relational model and stored in Oracle 9i relation database management system. Its web interface was constructed by using JSP scripts running on the Tom Cat web server, through which users have supervisory access. Java Servlets and JavaBeans were used to mediate interaction between clients and the database.

## Utility

LocustDB provides an interactive and user-friendly web interface for retrieving sequences and performing sequence alignment along with useful functional annotations. The main page includes the following interface: home, about, data, search, tool, and other accessory parts. Once clicking on the data icon, users can enter any part of the data modules: unigene model, its annotation and orthologous genes from comparative analysis with those of other insect species, enabling users to have a comprehensive overview of the stored data. Search engine is the entry point to the database, including both simple and advanced search modes. LocustDB hosts an online BLAST server for sequence-based search that yields sequence alignment, score, identity, E-value. And annotations of the corresponding homologous genes can be visualized simultaneously.

Upon clicking the search icon, users are presented with the advanced search interface of the database. The query starts with annotation and other basic analysis result of unigenes. Unigene and EST sequences from corresponding assemblies can be obtained individually or directly downloaded in bulk from the data module. For EST search, users can identify a unigene and its ESTs by inputting EST name or ID. For unigene search, users can enter a unigene name or annotation keywords, and detailed information, such as ORF length, GC content, EST linkage, unigene alignment, and unigene annotations will be presented in a result page. Hyperlinks provide as cross references for browsing definitions and associated components (such as KEGG pathway map, InterPro annotation, GO annotation, phylogenetic analysis of COG, and primary BLAST results). Users may search for keywords of function ontology, such as gene ontology number or terms, to find putative genes that possess specific functions as well as orthologous genes in other organisms. Alternatively, clients can choose appropriate definitions from public databases, including NCBI_NR, NCBI_NT, and Swiss_Prot. Links between the best BLAST hit to all unigenes and public databases were also established. A summary of BLAST hits and sequence alignment information from every BLAST analysis can be obtained upon clicking the link button. Furthermore, users can check for homologous genes between locust and the other invertebrate species whose genomic data are publicly available, through hyperlinks to these databases for tracing detailed information.

## Discussion and conclusion

The current aim of constructing LocustDB is to provide a catalog of genes expressed in the locust tissues and cells according to anatomic and phenotypic features to promote molecular entomology research. It will be modified frequently to serve as a framework for incorporating new genomic and proteomic data from the locust itself as well as other orthopteran and hemimetabolous insects. The database will also be updated for new versions with new data and biological information collected from the relevant literature in an ongoing effort. As a note for future development of this database, we plan to transform LocustDB into an integrated knowledgebase hosting information from genomic, biology, and ecology studies on the locust as well as other insects.

## Availability and requirements

LocustDB is maintained at the Beijing Genomics Institute and Institute of Zoology, Chinese Academy of Science. It is freely available at  by using web browsers. An e-mail message addressed to lkang@ioz.ac.cn may also be used for comments, corrections, and data submission. This database is freely available for download in the download entry.

## Authors' contributions

ZM carried out the data collection, test procedures, drafted the manuscript and also participated in the design.

LK and JY coordinated and supervised the whole project, suggesting the general direction and innovative features of the database and giving final approval of the version to be published.

All authors have read and approved the final manuscript.
